# Clinical and psychosocial determinants of oral handicap in systemic sclerosis: a cross-sectional study

**DOI:** 10.1007/s00296-026-06198-x

**Published:** 2026-06-22

**Authors:** Merve Erdoğan Soysal, Ipek Turk, Ayşegül Özdoğan Bircan, Metehan Soysal

**Affiliations:** 1 Department of Internal Medicine, Faculty of Medicine, Cukurova University, Adana, Turkey; 2 Division of Rheumatology, Department of Internal Medicine, Faculty of Medicine, Cukurova University, Adana, Turkey

**Keywords:** Systemic sclerosis, Microstomia, Quality of life, Anxiety, Depression, Patient reported outcome measures

## Abstract

To examine the relationship between oral handicap, clinical characteristics, psychosocial status, and quality of life in patients with systemic sclerosis (SSc). This cross-sectional study included 99 patients with SSc. Oral handicap was assessed using the Mouth Handicap in Systemic Sclerosis Scale (MHISS). Social appearance anxiety, anxiety/depression symptoms, resilience, and quality of life were evaluated using the Social Appearance Anxiety Scale, Hospital Anxiety and Depression Scale, Brief Resilience Scale, and World Health Organization Quality of Life–BREF (WHOQOL-BREF), respectively. Statistical and hierarchical multiple regression analyses were also performed. Oral handicap was associated with female sex (rho = 0.220, p = 0.029) and diffuse cutaneous SSc subtype (rho =  − 0.482, p < 0.001). Oral handicap showed strong positive correlations with the facial and total modified Rodnan Skin Score (rho = 0.851 and rho = 0.737; both p < 0.001). Positive associations were also observed for gastrointestinal (GI) involvement, calcinosis, telangiectasia, Scl-70 positivity, and digital ulcers (p < 0.002 for all). MHISS scores were positively correlated with anxiety, depression, and social appearance anxiety, and negatively correlated with all WHOQOL-BREF domains (p < 0.001 for all). In the regression analyses, mRSS (β = 0.484, p < 0.001), GI involvement (β = 0.210, p = 0.006), and anxiety scores (β = 0.183, p = 0.036) remained independently associated with oral impairment. The clinical model explained an additional 54% of variance, with a further 5% contributed by psychosocial variables. Oral impairment in SSc reflects both clinical disease burden and psychosocial distress, underscoring its multifactorial nature.

**Supplementary Information:**

The online version contains supplementary material available at 10.1007/s00296-026-06198-x.

## Introduction

Systemic sclerosis (SSc) is a rare connective tissue disease whose etiology has not yet been fully elucidated. It affects the skin and various internal organ systems, and is characterized by vasculopathy, chronic inflammatory processes, and progressive fibrosis [1, 2]. Beyond the well-known cutaneous and visceral features of SSc, there are also significant orofacial changes that are often overshadowed by more prominent systemic findings in the early diagnosis and management process and may therefore be overlooked [3].

The term microstomia refers to a constricted mouth opening, characterized by a distance between the upper and lower incisors of less than 40 mm. This is a prevalent orofacial finding, with a strong association to SSc, observed in 60–91.4% of patients [4]. Characterized by fibrotic changes in the perioral soft tissues, this condition exerts a substantial detrimental influence on the individual’s quality of life, given the multifaceted consequences it involves. These include, among others, deterioration in oral and dental health, difficulties in mastication, nutritional problems, and reduced participation in daily social life. Reduced mouth opening can result in complications during anesthetic procedures and respiratory function testing [5].

SSc has been shown to have a detrimental effect on both physical and psychological well-being; for instance, alterations in facial expressions can severely impact an individual’s mental health. The prevalence of depressive symptoms is notably high in this patient group, and both subjective health perception and disease-related quality of life are generally low [6, 7]. The decline in subjective health status is closely related to dietary restrictions associated with problems affecting mouth opening, chewing, and swallowing [6]. The development of malnutrition in SSc is significantly influenced by microstomia; conversely, malnutrition can be both a cause and a consequence of depression in these patients [8].

Although previous studies on SSc have mainly focused on limitations in mouth opening or MHISS scores, the combined clinical and psychosocial determinants of oral impairment remain insufficiently explored. Therefore, this study aimed to evaluate oral impairment in patients with SSc and examine its associations with clinical characteristics, psychological resilience, social appearance anxiety, mood symptoms and quality of life, adopting a more patient-centred approach. Understanding these associations could help to facilitate the earlier recognition of oral disability and inform the development of more effective, multidisciplinary rehabilitation strategies for patients with SSc.

## Materials and methods

### Study population and design

The present study population comprised a total of 99 patients diagnosed with SSc according to the American College of Rheumatology/European League Against Rheumatism (ACR/EULAR) 2013 classification criteria, who applied to our rheumatology clinic between October 2023 and January 2024 [9]. Exclusion criteria included being younger than 18 years, having SSc overlap syndrome, refusing to participate in the study, the presence of serious neurological or psychiatric disease that would prevent the application of questionnaires, undergoing active malignancy treatment, the presence of scars on the face and neck area due to trauma, medical intervention history, or any other cause, and pregnancy.

This research was approved by the Cukurova University Human Research Ethics Committee (dated: 13.10.2023, no: 137. All participants were adequately informed about the purpose and scope of the study, and written informed consent was obtained from each participant prior to enrolment. The principles of the Declaration of Helsinki were observed throughout the process. The study was conducted in accordance with the Strengthening the Reporting of Observational Studies in Epidemiology (STROBE) guidelines [10].

### Clinical assessment and study measures

The sociodemographic characteristics of the participants (i.e. age, sex, education, and marital status), smoking habits, and comorbidities were recorded. They were categorized into SSc subtypes based on the distribution of skin involvement: limited (confined to distal areas) and diffuse (involving proximal areas as well) [11]. Disease duration was defined as the time from the onset of non-Raynaud’s symptoms to study inclusion.

Skin involvement was evaluated using the modified Rodnan Skin Score (mRSS). Skin thickness across 17 anatomical sites, including the face, was scored on a scale from 0 (normal) to 3 (severe thickening) [12]. Facial skin thickness was graded across four levels: none, mild, moderate, and severe. Additional clinical features evaluated included calcinosis, telangiectasia, arthritis, digital ulcers, and a weight loss exceeding 5% of body weight within the preceding six months. Data regarding SSc therapies over the past six months and iloprost treatment within the preceding year were also collected.

Clinical and laboratory data retrieved from the hospital database were used to evaluate pulmonary involvement. Interstitial lung disease (ILD) was identified based on characteristic patterns observed on high-resolution computed tomography (HRCT) supported by restrictive findings on pulmonary function tests [5, 13]. Systolic pulmonary artery pressure (sPAP) was estimated by Doppler echocardiography, while forced vital capacity (FVC) and diffusing capacity of the lungs for carbon monoxide (DLCO) were also recorded. When available, mean PAP (mPAP) values measured by right heart catheterisation (RHC) were included. Pulmonary hypertension was defined as a resting mPAP ≥ 25 mmHg with a pulmonary arterial wedge pressure (PAWP) ≤ 15 mmHg on RHC, or an estimated sPAP > 40 mmHg on Doppler echocardiography [14].

Cardiac involvement, positive endomyocardial biopsy results, cardiac magnetic resonance imaging (MRI) showing characteristics of cardiac disease associated with SSc, or the presence of cardiac abnormalities like arrhythmia, conduction abnormalities, pericardial effusion, or ventricular systolic/diastolic dysfunction are all signs of SSc-related cardiac involvement [15].

Gastrointestinal (GI) involvement was defined by the presence of one or more of the following manifestations, including gastroesophageal reflux disease (GERD) (e.g., hoarseness, oropharyngeal dysphagia, reflux, and heartburn), intestinal dysmotility (e.g., abdominal tightness and bloating, prolonged constipation, or episodes of diarrhea), lower esophageal sphincter (LES) dysfunction, esophageal dysmotility (e.g., distal dysphagia), and anorectal dysfunction [16].

Scleroderma renal crisis (SRC) is characterized by acute kidney injury (AKI), microangiopathic hemolytic anemia (MAHA), thrombocytopenia, target organ dysfunction and the presence of kidney-specific vasculopathic pathologies, as evidenced by clinical and laboratory findings such as a significant increase in serum creatinine levels or a decrease in urine output within a short period of time [17].

Data on antinuclear antibody (ANA), antitopoisomerase I (anti-Scl-70), and anticentromere antibody (ACA) positivity were obtained from laboratory test results and recorded.

The European Scleroderma Trials and Research Group Activity Index (EUSTAR-AI) was used to assess disease activity [18].

### Assessment instruments

#### Mouth Handicap in Systemic Sclerosis (MHISS) Scale

The MHISS was created to evaluate oral function deficits and related challenges in individuals with SSc [19]. The Turkish version of the MHISS (MHISS-T) has demonstrated good reliability and validity in the Turkish population [20]. The scale comprises 12 items, with scores ranging from 0 (never) to 4 (always). The overall score ranges from 0 to 48, where higher numbers signify a higher level of functional impairment. The MHISS-T comprises three subscales: mouth opening (items 1, 3, 4, 5, and 6), dry mouth (items 2, 7, 8, 9, and 10), and esthetic concerns (items 11 and 12).

#### Social Appearance Anxiety Scale (SAAS)

The SAAS is a 16-item self-report questionnaire designed to evaluate the behavioral, emotional, and cognitive components of anxiety related to body image physical appearance. Responses are scored on a 5-point Likert scale, with overall scores ranging from 16 to 80 and each item rated from 1 (not at all true) to 5 (totally true). Higher scores indicate a greater severity of social appearance anxiety [21]. The validity and reliability of the Turkish version of the scale were previously established [22].

#### World Health Organization Quality of Life Short Form-BREF (WHOQOL-BREF)

The WHOQOL-BREF was designed as a condensed version of the original 100-item form and contains 26 questions to gauge an individual’s subjective assessments of their quality of life. General health (questions 1 and 2), physical health (questions 3, 4, 10, and 15–18), psychological health (questions 5–7, 11, 19, and 26), social relationships (questions 20–22), and environmental factors (questions 8–9, 12–14, and 23–25) are all covered in the scale, which evaluates participants’ conditions over the previous two weeks. An increased quality of life is indicated by higher scores [23]. The Turkish version of the scale has also been validated [24].

#### Hospital Anxiety and Depression Scale (HADS)

The HADS is a self-report questionnaire that is used to screen for and grade the severity of anxiety and depression. It has two subscales: one for depression and one for anxiety. Each item is rated on a scale between 0 and 3. Items with a forward score range from 0 to 3, and those with a reverse score go from 3 to 0. Items 1, 3, 5, 7, 9, 11, and 13 comprise the anxiety subscale score, while items 2, 4, 6, 8, 10, 12, and 14 comprise the depression subscale score. Each subscale has a total score between 0 and 21. A score of 0–7 is normal. A score of 8–10 indicates a possible mood disorder, while a score of 11 or above suggests a high probability of a mood disorder [25]. Original and Turkish validation studies were previously carried out [26].

#### Brief Resilience Scale (BRS)

The BRS is a measure that focuses on assessing an individual’s ability to recover, adapt, and return to their previous level of functioning. This six-item, 5-point Likert-type scale is scored from “Strongly disagree” (1) to “Strongly agree” (5). Items 2, 4, and 6 are reverse-coded. High total scores obtained after reverse coding indicate a higher level of psychological resilience [27]. The scale’s original development study and Turkish validity and reliability studies have been established [28].

All scores, scales, and questionnaires used in the study were administered in accordance with their original definitions and validated Turkish versions, while considering current recommendations for survey studies and cross-cultural adaptation [29, 30]. Where applicable, the necessary permissions for their use were obtained.

### Statistical analysis

Statistical analyses were performed using IBM SPSS Statistics for Windows 27.0 (IBM Corp., Armonk, NY, USA). Data distribution was evaluated using the Kolmogorov–Smirnov test, inspection of skewness and kurtosis values, histograms, and Q–Q plots. Continuous variables were presented as the mean ± standard deviation (SD) or median (25th–75th percentile), as appropriate according to their distribution, whereas categorical variables were expressed as numbers and percentages [n (%)]. To examine the relationship between oral impairment and clinical, demographic, and psychosocial characteristics, Spearman’s rank correlation coefficients (rho) were calculated between the MHISS-T score and all study variables. Dichotomous categorical variables were coded numerically prior to correlation analyses (0 = absence, 1 = presence). Sex and disease subtype variables were also numerically coded as specified in the table footnotes. Positive correlation coefficients were interpreted as indicating that the presence of the related clinical feature was associated with greater oral handicap severity.

Three-step hierarchical multiple regression analysis was performed to identify independent predictors of MHISS-T scores. Candidate variables were selected based on the results of the correlation analysis, clinical relevance, and intercorrelation criteria. In addition, the number of predictors included in the final model was restricted according to the sample size to minimize the risk of overfitting. Variables showing significant associations with the MHISS-T, considered clinically relevant determinants, and having intercorrelations below 0.70 were included in the model. Variables with high intercorrelation (r > 0.70), such as the EUSTAR-AI and HADS-depression subscale, were excluded to avoid multicollinearity. Demographic covariates (age and sex) were entered in Model 1; clinical variables (SSc subtype, mRSS, GI involvement, and presence of digital ulcers) were added in Model 2; and psychosocial variables (HADS-anxiety score and BRS score) were included in Model 3. Before conducting the regression analyses, model assumptions were examined. The normality and homoscedasticity of residuals were assessed using histogram and normal probability (Q–Q) plots as well as scatterplots of standardized residuals. Linearity was evaluated by inspecting partial regression plots. Multicollinearity was assessed using tolerance and variance inflation factor (VIF) statistics, with tolerance values > 0.20 and VIF values < 5 considered acceptable.

A two-tailed p-value of < 0.05 was considered statistically significant.

## Results

Data from 99 patients with SSc enrolled in the study were analyzed, and their demographic and clinical characteristics of the patients are summarized in Table 1.

**Table 1:** Demographic and clinical characteristics of the patients

Variable	Value
Age, (years)^b^	56.0 ± 11.9
Sex, (Female)^a^	90 (90.9)
BMI, (kg/m^2^)^b^	26.8 ± 5.7
Education status^a^ Illiterate Primary/secondary school High school University/graduate	23 (23.2) 49 (49.5) 17 (17.2) 10 (10.1)
Marital status^a^ Married Single	81 (81.8) 18 (18.2)
Smoking^a^ Non-smoker Active smoker Ex-smoker	69 (69.7) 11 (11.1) 19 (19.2)
Package/year, n^c^	10.0 (6.0 – 32.5)
Disease duration, (years)^b^	8.7 ± 6.7
SSc subtype^a^ Diffuse Limited	51 (51.5) 48 (48.5)
Antinuclear antibodies^a^	91 (91.9)
Anti–topoisomerase I antibody (Scl-70)^a^	54 (54.5)
Anticentromere antibody^a^	26 (26.3)
MHISS-T^c^	26.0 (7.0–38.0)
Social Appearance Anxiety Scale^b^	43.1 ± 18.8
WHOQOL-BREF general health (items 1–2)^b^	43.9 ± 25.5
WHOQOL-BREF physical health (items 3, 4, 10, 15, 16, 17, 18)^b^	44.2 ± 22.2
WHOQOL-BREF psychological health (items 5, 6, 7, 11, 19, 26)^b^	58.1 ± 20.7
WHOQOL-BREF social relationships (items 20, 21, 22)^b^	53.3 ± 24.0
WHOQOL-BREF environment (items 8, 9, 12, 13, 14, 23, 24, 25)^b^	56.0 ± 19.7
HADS-anxiety category^a^ Normal Borderline Abnormal	48 (48.5) 18 (18.2) 33 (33.3)
HADS-anxiety score^b^	8.1 ± 5.1
HADS-depression category^a^ Normal Borderline Abnormal	49 (49.5) 24 (24.2) 26 (26.3)
HADS-depression score^b^	6.8 ± 4.6
Brief resilience scale (total score)^c^	22.0 (13.0–26.0)
EUSTAR activity index^b^	4.1 ± 2.9
Modified Rodnan Skin Score – face^a^ Normal Mild thickening Moderate thickening Severe thickening	18 (18.2) 30 (30.3) 30 (30.3) 21 (21.2)
Modified Rodnan Skin Score-total score^b^	19.2 ± 10.2
Medications^a^ Cyclophosphamide Mycophenolate mofetil DMARD Azathioprine Rituximab DMARD + mycophenolate mofetil/azathioprine DMARD + mycophenolate mofetil/azathioprine + nintedanib	0 (0.0) 29 (29.3) 32 (32.3) 19 (19.2) 0 (0.0) 9 (9.1) 10 (10.1)
Iloprost^a^	32 (32.3)
Clinical manifestations^a^ Telangiectasia Calcinosis Weight loss Arthritis Digital ulcer	65 (65.7) 45 (45.5) 15 (15.2) 9 (9.1) 47 (47.5)
Interstitial lung disease^a^	75 (75.8)
Pulmonary arterial hypertension^a^	16 (16.2)
Cardiovascular involvement^a^	49 (49.5)
Gastrointestinal involvement^a^	70 (70.7)
Scleroderma renal crisis^a^	0 (0.0)
FVC-predicted (%)^b^	84.3 ± 25.8
DLCO-predicted (%)^b^	63.6 ± 19.7

*BMI* Body Mass Index, *MHISS-T* The Mouth Handicap in Systemic Sclerosis-Turkish version, *WHOQOL-BREF *World Health Organization Quality of Life-BREF Questionnaire; *HADS* Hospital Anxiety and Depression Scale, *DMARD* Disease-Modifying Antirheumatic Drug, *FVC* Forced Vital Capacity; *DLCO* Diffusing Capacity of the Lung for Carbon Monoxide

^a^n (%)

^b^Mean ± standard deviation

^c^Median (25–75%)

The statistical analysis revealed a weak but significant association with sex (rho = 0.220, p = 0.029), with females exhibiting slightly higher MHISS-T scores. The analysis also showed a moderate negative association with SSc subtype (rho = − 0.482, p < 0.001). Very strong and strong positive correlations were found with facial mRSS (rho = 0.851, p < 0.001) and total mRSS (rho = 0.737, p < 0.001), demonstrating that increasing skin thickening was strongly associated with reduced oral function. Moderate positive associations were observed for Scl-70 positivity (rho = 0.317, p = 0.001) and the presence of digital ulcers (rho = 0.301, p = 0.002). In addition, telangiectasia (rho = 0.505, p < 0.001), calcinosis (rho = 0.538, p < 0.001), GI involvement (rho = 0.519, p < 0.001), and iloprost therapy (rho = 0.381, p < 0.001) showed moderate to strong associations with higher MHISS-T scores. Pulmonary function parameters, including FVC% (rho = − 0.265, p = 0.018) and DLCO% (rho = − 0.341, p = 0.003), demonstrated small-to-moderate negative correlations, suggesting that reduced pulmonary capacity was associated with greater oral impairment.

Hierarchical multiple regression analysis revealed that in Model 1, sex was significantly associated with MHISS-T (β = 0.218, p = 0.032), whereas age was not (p = 0.849). This model explained 3% of the variance in MHISS-T scores (adjusted R^2^ = 0.03). In Model 2, mRSS was a strong independent predictor of MHISS-T (β = 0.490, p < 0.001), while GI involvement was also significantly associated with higher MHISS-T scores (β = 0.250, p = 0.002). SSc subtype and the presence of digital ulcers were not significantly associated with MHISS-T. The inclusion of clinical variables increased the explained variance by 54% (ΔR^2^ = 0.54). In Model 3, mRSS (β = 0.484, p < 0.001) and GI involvement (β = 0.210, p = 0.006) remained significant predictors. In addition, higher anxiety scores were independently associated with increased MHISS-T (β = 0.183, p = 0.036). BRS scores were not significantly associated with MHISS-T (p = 0.409). The inclusion of psychosocial variables explained an additional 5% of the variance (ΔR^2^ = 0.05). Overall, the final model indicated that mRSS, GI involvement, and anxiety scores were independently associated with MHISS-T, together accounting for a substantial proportion of the variance in oral impairment (Tables 2, 3, 4).

**Table 2:** Correlation of MHISS-T scores with demographic, clinical, and laboratory parameters

	MHISS-T	
	Spearman’s rho	p-value
Age	− 0.037	0.720
Sex	0.220	0.029
BMI	− 0.159	0.117
Education level	0.036	0.724
Pack-years	− 0.202	0.294
Disease duration	0.147	0.145
SSc subtype	− 0.482	< 0.001
Antinuclear antibody (ANA)	0.179	0.077
Anti-topoisomerase I (anti-Scl-70)	0.317	0.001
Anticentromere antibody (ACA)	− 0.279	0.006
Facial mRSS	0.851	< 0.001
Total mRSS	0.737	< 0.001
Iloprost treatment	0.381	< 0.001
Telangiectasia	0.505	< 0.001
Calcinosis	0.538	< 0.001
Weight loss (> 5% in 6 months)	0.268	0.007
Arthritis	0.288	0.004
Digital ulcers	0.301	0.002
Interstitial lung disease	0.201	0.047
Pulmonary arterial hypertension	0.139	0.171
Cardiovascular involvement	0.117	0.247
Gastrointestinal involvement	0.519	< 0.001
Scleroderma renal crisis	NA	NA
FVC (% predicted)	− 0.265	0.018
DLCO (% predicted)	− 0.341	0.003

Sex was coded as male = 0 and female = 1; SSc subtype was coded as diffuse = 0 and limited = 1; education level ranged from 0 (illiterate) to 4 (university)

*BMI* Body Mass Index, *mRSS* modified Rodnan Skin Score, *NA* not applicable, *FVC* Forced Vital Capacity, *DLCO* Diffusing Capacity of the Lung for Carbon Monoxide

**Table 3:** Association of psychosocial measures with the MHISS-T score in patients with systemic sclerosis

	MHISS-T	
	Spearman’s rho	p-value
Social Appearance Anxiety Scale	0.612	< 0.001
WHOQOL-BREF general health	− 0.470	< 0.001
WHOQOL-BREF physical health	− 0.604	< 0.001
WHOQOL-BREF psychological health	− 0.431	< 0.001
WHOQOL-BREF social relationships	− 0.385	< 0.001
WHOQOL-BREF environment	− 0.475	< 0.001
HADS-anxiety score	0.387	< 0.001
HADS-depression score	0.480	< 0.001
Brief resilience scale (total score)	− 0.381	< 0.001
EUSTAR activity index	0.636	< 0.001

*MHISS-T* The Mouth Handicap in Systemic Sclerosis-Turkish version, *WHOQOL-BREF* World Health Organization Quality of Life-BREF Questionnaire, *HADS* Hospital Anxiety and Depression Scale

**Table 4:** Hierarchical multiple regression models examining demographic, clinical, and psychosocial predictors of MHISS-T scores

	B (95% CI)	Beta	p-value
*Model 1: Confounders*			
Age Sex	− 0.02 (− 0.28 to 0.23) 11.38 (1.0 to 21.7)	-0.019 0.182	0.849 0.032
Note	Constant (SE) = 3.3 (12.7) Adjusted R^2^ = 0.03		
*Model 2: Clinical variables*			
Age Sex SSc subtype mRSS Gastrointestinal involment Digital ulcer	0.07 (− 0.10 to 0.24) 1.93 (− 5.31 to 9.18) − 4.32 (− 9.31 to 0.66) 0.73 (0.44 to 1.01) 8.24 (3.16 to 13.32) 2.84 (− 1.65 to 7.33)	0.056 0.037 − 0.144 0.490 0.250 0.094	0.416 0.597 0.088 < 0.001 0.002 0.213
Note	Constant (SE) = 23.47 (12.91) Delta R^2^ = 0.54		
*Model 3: Clinical and psychosocial variables*			
Age Sex SSc subtype mRSS Gastrointestinal involment Digital ulcer HADS-anxiety score Brief resilience scale	0.08 (− 0.09 to 0.25) 1.74 (0.62 to − 5.14) − 3.70 (− 8.43 to 1.03) 0.72 (0.45 to 0.99) 6.93 (2.05 to 11.80) 1.66 (− 2.64 to 5.96) 0.55 (0.04 to 1.06) − 0.16 (− 0.54 to 0.22)	0.063 0.033 − 0.123 0.484 0.210 0.055 0.183 − 0.073	0.348 0.616 0.123 < 0.001 0.006 0.445 0.036 0.409
Note	Constant (SE) = 17.73 (13.96) Delta R^2^ = 0.05		

Sex was coded as male = 0 and female = 1. SSc subtype was coded as diffuse = 0 and limited = 1

*B* Unstandardized Coefficients, *Beta* Standardized Coefficients, *SE* Standard error, *SSc* Systemic Sclerosis, *HADS* Hospital Anxiety and Depression Scale, *mRSS* Modified Rodnan Skin Score-total score

## Discussion

The present investigation examined the relationship between oral impairment, as assessed by the MHISS-T, and disease characteristics, clinical findings, and psychosocial factors in individuals with SSc. In addition to statistical analyses, hierarchical multiple regression models were applied to identify independent predictors of oral impairment and to evaluate the additional contribution of clinical and psychosocial variables.

According to the present findings, oral handicap was weakly but significantly associated with female sex but showed no relationship with age or body mass index (BMI). In a study conducted within a Jordanian population, mouth opening was significantly associated with sex but not with age; however, it was significantly correlated with height and body weight [31]. Similarly, a study from North India reported that females had significantly smaller mouth apertures than males. Additionally, mouth opening was shown to decrease with age in both sexes, a trend linked to a longer mandibular structure and stronger masticatory muscles in men [32].

The results show that oral handicap and facial and total mRSS are significantly and positively correlated. Very strong and strong positive correlations were observed with facial mRSS and total mRSS, respectively, indicating that increasing skin thickening was strongly associated with greater patient-reported oral handicap. These findings are in line with a previous study reporting a significant inverse relationship between interincisal distance and total mRSS in patients with SSc [5]. Consistent with the results herein, the Canadian Systemic Sclerosis Oral Health Study III also revealed a significant negative relationship between mRSS and mouth opening [33].

Anti-Scl-70 antibody positivity was moderately associated with greater oral handicap, whereas SSc subtype also showed a moderate association with the condition, with more pronounced involvement in patients with diffuse cutaneous SSc (dcSSc). These findings align with those from a study on 382 SSc patients, which found that orofacial involvement, as assessed by MHISS and maximum mouth opening (MMO) measurements, was more pronounced in patients with anti-Scl-70 positivity and milder in those with ACA positivity, and was associated with widespread skin involvement [34]. Another study involving 80 SSc patients (55 with dcSSc) reported that the interincisal distance was lower in anti-Scl-70 positivite patients and higher in ACA positivite patients [5]. Taken together, these findings suggest that the autoantibody profile and disease subtype may play an important role in determining the degree of orofacial involvement through the extent of skin fibrosis. This implies that the systemic fibrotic burden may be reflected in oral handicap.

Among our significant findings, positive associations were observed between oral handicap and telangiectasia, calcinosis, GI involvement, digital ulcers, and iloprost therapy. Calcinosis develops in SSc alongside chronic inflammation, recurrent tissue damage, and vascular dysfunction [35, 36]. These findings suggest that patient-reported oral handicap may be associated with vascular and fibrotic disease patterns. The association with GI involvement further indicates that oral handicap may accompany a broader functional burden affecting nutrition, swallowing, and daily functioning in SSc. Khidir et al. [34] reported that peripheral vascular involvement and reduced MMO were significantly associated, while interincisal distance (IID) and the occurrence of digital ulcers showed no significant interaction in a study by Turk et al. [5]. Since different studies have addressed peripheral vascular involvement using different definitions and measurement techniques, methodological differences may explain this disparity in results. A review of orofacial involvement in SSc showed that microstomia is the most common finding and that telangiectasia is the most frequently associated skin feature, which supports our findings [3]. In addition, lower FVC% and DLCO% were associated with higher MHISS-T scores, further supporting a link between oral impairment and overall disease severity.

The current study highlights that oral aperture limitation may affect not only patients' physical functions but also broader psychosocial domains such as social appearance perception, psychological well-being, and social participation, alongside the clinical burden of SSc. In this context, the finding that MHISS-T scores are positively associated with social appearance anxiety suggests that increasing oral deterioration may heighten patients’ concerns about appearance-related evaluation. Indeed, in a review by Gholizadeh et al. addressing the measurement and management of appearance concerns in SSc, it was emphasized that visible orofacial changes create a significant psychosocial burden for patients and that there is a need to develop interventions in this area [37]. Similarly, Jewett et al. examined the sociodemographic and disease-related determinants of body image distress in 489 patients with SSc included in the Canadian Scleroderma Research Group registry system and demonstrated that appearance-related anxiety is a meaningful psychosocial outcome domain alongside clinical burden [38]. These data confirm that the relationship between oral restriction and social appearance anxiety in our study is consistent with the literature.

The consistently negative correlations between MHISS-T scores and all subscales of the WHOQOL-BREF indicate that oral functional limitations adversely affect multiple dimensions of quality of life in individuals with SSc. A review in 2026 further emphasized that reduced oral aperture and other oral manifestations in SSc may impair mastication, oral hygiene, speech, and social interactions, thus substantially affecting quality of life and daily functioning [39]. In a recently published mixed-methods study, a survey was administered to 1853 patients with systemic autoimmune rheumatic diseases, and in-depth interviews were conducted with 34 patients. In this cohort, patients with SSc constituted 3.4% of the survey group and 9% of the interview group. The researchers vividly described how patients’ social participation had decreased significantly and their living space had narrowed due to the burden of chronic disease, using the phrase “my world has shrunk” [40]. In a prospective cross-sectional recently published study, capillaroscopic patterns in SSc were evaluated in conjunction with key clinical domains and patient-reported outcome measures; it was reported that more advanced capillaroscopic patterns were associated with poorer quality of life, increased functional impairment, and a higher patient-perceived disease burden. These findings support the role of patient-reported outcome measures in complementing clinical assessment to reveal the broader impacts of the vascular and fibrotic disease burden in SSc [41]. Likewise, a recent review by Aboabat emphasized that quality of life and patient-reported outcomes should be among the primary treatment goals in the management of SSc [42]. These data support the notion that the effects of oral impairment on quality of life should not be overlooked in clinical assessment.

MHISS-T scores in the present study were associated with symptoms of anxiety and depression, with anxiety remaining an independent predictor in the multivariable model, suggesting that oral manifestations may be closely linked to the emotional state of patients with SSc. Oral involvement affecting fundamental areas such as speech, eating, and social interaction may be a factor that increases emotional burden. This result is consistent with a comprehensive systematic review published in 2023 reporting that mood and anxiety disorders are common in patients with SSc [43]. In this context, a recent study showing that discrepancies between patient and physician global assessments are common, reporting that patient assessments are more influenced by areas based on patient experience, such as pain, loss of physical function, and disease burden, whereas physician assessments are more closely associated with traditional clinical indicators. These findings support the view that patient-reported outcome measures play a complementary role in identifying aspects of the disease burden in SSc that are not fully reflected in clinical assessments [44].

The current study showed that psychological resilience was negatively associated with oral impairment; however, this relationship did not remain significant in the multivariable model. Resilience is an important psychosocial determinant that reflects an individual's capacity to adapt to physical limitations and disease burden in chronic rheumatological diseases. In this context, a cross-sectional study conducted by Neyer et al., utilizing the SPIN cohort, emphasized that resilience is closely related to positive mental well-being in patients with SSc and indicated that resilience could be an important target in terms of patient-centered outcomes [45]. In situations accompanied by oral restrictions, reduced resilience may contribute to a greater functional burden and coping difficulties. Indeed, a study by Uluışık et al. supported this interactive process by showing that kinesiophobia is closely related to physical performance in patients with SSc [46].

Overall, the hierarchical regression model substantially strengthened the interpretation of our findings by distinguishing the independent determinants of oral impairment from bivariate associations. Of all the evaluated variables, skin thickening was found to be the most robust predictor, highlighting the pivotal role of fibrosis in the development of orofacial impairment in SSc. GI involvement also retained independent significance, indicating that oral impairment may reflect a broader functional impairment that extends beyond the oral cavity. Additionally, anxiety symptoms provided incremental explanatory value, indicating that emotional distress contributes to oral impairment beyond clinical disease severity alone. Taken together, these findings support a multidimensional model in which oral impairment in SSc is driven by organ involvement and psychosocial burden.

This study had several limitations. Due to its cross-sectional design, the observed associations cannot be interpreted as causal relationships. In addition, the single-center setting and a modest sample size may limit the generalizability of the findings. The possibility of selection bias related to the application-based recruitment should also be acknowledged. Furthermore, the lack of objective measurements for mouth opening, such as maximum mouth opening or interincisal distance, was a significant limitation. In addition, dental/oral examination findings, salivary function tests, and oral health indices were not assessed. As oral handicap was assessed solely using the patient-reported MHISS-T, it was not possible to evaluate the relationship between self-reported oral functional limitations and objectively measured mouth opening or other objective oral health parameters. Therefore, the current findings should be interpreted as reflecting patients’ perceived limitations in oral function rather than objective measurements. Future studies combining the MHISS-T with objective orofacial measurements, dental/oral assessments, salivary function tests, and oral health indices may provide a more comprehensive assessment of orofacial involvement in SSc. Nevertheless, the use of validated instruments and the comprehensive evaluation of both clinical and psychosocial factors enhance the robustness of the study. A major strength of this work was its multidimensional approach to orofacial impairment in SSc, incorporating not only clinical characteristics but also patient-centered domains such as social appearance concerns, quality of life, depression, anxiety symptoms, and resilience. The integration of detailed clinical data with oral impairment assessment within a single analytical framework strengthens the clinical relevance of the findings. Moreover, the application of hierarchical regression analyses allowed the identification of independent predictors of oral impairment and demonstrated the incremental contribution of clinical and psychosocial variables.

In conclusion, this study demonstrated that patient-reported oral impairment in SSc is significantly associated with multiple patient-reported outcomes, including social appearance concerns, quality of life, emotional status, and psychological resilience. Hierarchical regression analyses further revealed that skin thickening, GI involvement, and anxiety symptoms were independent predictors of MHISS-T scores. These findings suggest that the impact of orofacial involvement on disease burden reflects both clinical severity and psychosocial well-being. However, because oral impairment was assessed using the patient-reported MHISS-T without objective orofacial measurements, the results should be interpreted as reflecting perceived limitations in oral function rather than objective measurements. Therefore, a comprehensive assessment of oral involvement, including both patient-reported and objective measures, may be important in the follow-up of patients with SSc.

## Supplementary Information

Below is the link to the electronic supplementary material.

Supplementary file1 (PDF 1290 KB)

## Data Availability

The datasets gathered during the preparation of this manuscript are available from the corresponding author upon reasonable request.

## Declarations

### Disclaimer

The authors confirm that this article, including the text and graphics, has not been copied in whole or in part, published elsewhere, or generated by AI. This work has not previously been presented as a conference abstract or published in any abstract book.
